# Christie, Atkins, Munch–Peterson (CAMP) Negative Listeria monocytogenes Meningitis in Neonates: Report of Two Cases

**DOI:** 10.7759/cureus.55800

**Published:** 2024-03-08

**Authors:** Rahul Ranjan, Ankita Rai, Pooja Pandey, Raunak Bir, Priti Agarwal

**Affiliations:** 1 Department of Microbiology, Employees' State Insurance Corporation (ESIC) Medical College and Hospital, Faridabad, IND

**Keywords:** stillbirth, food borne, ampicillin, camp test, pregnancy, tumbling motility, preterm, abortions, meningitis, neonate

## Abstract

*Listeria monocytogenes*, a gram-positive bacillus and an intracellular pathogen, is an uncommon cause of illness in the general population. During pregnancy, a perinatal infection can lead to serious complications such as abortion, stillbirth, neonatal sepsis, and meningitis. We present two cases of neonatal meningitis caused by Christie, Atkins, Munch-Peterson (CAMP)-negative* Listeria monocytogenes*.

In the first case, a seven-day-old female term neonate delivered vaginally, presented with high-grade fever and refusal to feed. In view of the suspected late-onset sepsis, a septic workup, including cerebrospinal fluid analysis, was conducted. CSF culture reports obtained showed a growth consistent with *Listeria monocytogenes,* which was CAMP test negative and susceptible to the penicillin group of drugs, cotrimoxazole, erythromycin, and meropenem. The isolate was identified using matrix-assisted laser desorption ionization-time of flight mass spectrometry (MALDI-TOF MS) and confirmed by 16S rRNA sequencing. The blood culture was sterile. At 48 hours of admission, the neonate clinically deteriorated with fluctuation in oxygen saturation below 95% at room air. Thus, she was electively intubated and connected to the mechanical ventilator with appropriate settings. The antibiotics were upgraded to meropenem from the empirical antibiotic therapy. The neonate showed clinical improvement within the next 24 hours of initiating antibiotics according to culture susceptibility and was gradually weaned from the mechanical ventilator to continuous positive airway pressure (CPAP). After 24 hours, she was able to maintain normal saturation at room air.

In the second case, an 11-day-old low birth weight neonate, small for gestational age, was presented to the NICU with complaints of loose stools, fever, and refusal to feed for the past two days. In view of the suspected sepsis, relevant investigations were carried out while initiating empirical antibiotics IV piperacillin-tazobactam and IV amikacin for the neonate. Meanwhile, there was a dip in oxygen saturation noted on room air for the neonate and he/she was mechanically ventilated. The CSF culture grew *Listeria monocytogenes*,which* *was identified using MALDI-TOF MS and confirmed by 16S rRNA sequencing. The isolate tested negative for the CAMP test and was susceptible to ampicillin, penicillin, cotrimoxazole, erythromycin, and meropenem. The blood culture was sterile. The antibiotics were upgraded to meropenem from the empirical antibiotic therapy, the patient’s condition improved, and the baby was eventually discharged.

## Introduction

*Listeria monocytogenes*, a Gram-positive bacillus, is an intracellular pathogen that has been linked to several foodborne disease outbreaks over the past decade [1]. While *Listeria monocytogenes* is not commonly associated with illness in the general population, it becomes significant in specific patient groups. Immunosuppressant subsets of neonates, pregnant women, elderly (>65 years or so), transplant recipients, and individuals with impaired cell-mediated immunity. In these vulnerable groups, *Listeria* can cause life-threatening conditions such as bacteremia and meningoencephalitis [2]. *Listeria monocytogenes* has a particular predilection for the CNS and the placenta. Perinatal listeriosis, caused by *Listeria monocytogenes*, manifests as a clinical syndrome that includes abortion, stillbirth, neonatal sepsis, and meningitis [3]. The organisms are known to be transmitted by ingestion of foods such as unpasteurized milk, soft cheese, undercooked poultry, and unwashed raw vegetables. Although most human listeriosis appears to be foodborne, other modes of transmission occur including from mother to child trans-placentally or through an infected birth canal, cross-infection in neonatal nurseries, etc. [4]. In India, infections with *Listeria monocytogenes *remain largely undiagnosed and under-reported because of a lack of awareness and suspicion to promptly isolate and identify the organism [5]. The incubation period for listeriosis varies according to the clinical presentation of the disease ranging from 3 days to 70 days [6].

## Case presentation

We report two cases of Christie, Atkins, Munch-Peterson (CAMP) test-negative neonatal Listeria meningitis from a tertiary care hospital in Faridabad to re-emphasize the occurrence and identification of *Listeria monocytogenes* meningitis in the neonatal period.

Case 1

A newborn, full-term, vaginally delivered, female baby weighing 2.725 kg was admitted to the neonatal intensive care unit (NICU) of a tertiary care hospital on Day 7 of birth with complaints of dull activity, non-acceptance of feed, hyperthermia, and multiple episodes of apnea.

During the antenatal period, the mother had an uneventful course, with no reported prolonged rupture of membranes or foul-smelling amniotic fluid. Tests for HIV, hepatitis, toxoplasma, rubella, and herpes simplex types 1 and 2 using enzyme-linked immunosorbent assay (ELISA) all returned negative results. Both the husband and wife tested non-reactive for syphilis based on the venereal disease research laboratory (VDRL) test. The mother had no history of diabetes mellitus, smoking, alcohol consumption, or drug intake during the antenatal period.

During the examination, the patient exhibited a fever. Reflexes were diminished, and the umbilicus appeared healthy without any signs of focal neurological deficits or nuchal rigidity. No gross congenital anomalies were observed and there was no history of diarrhea.

The baby’s heart rate was 138 beats per minute, and the respiratory rate was 48 breaths per minute. Due to suspected meningitis, a lumbar puncture was performed, and both cerebrospinal fluid (CSF) and blood samples were sent for culture. The baby’s total leukocyte count was 17,230/cu mm with 70% neutrophils. Empirical treatment was initiated with IV cefotaxime (150 mg BD) and IV amikacin (40 mg OD).

**Figure 1:**
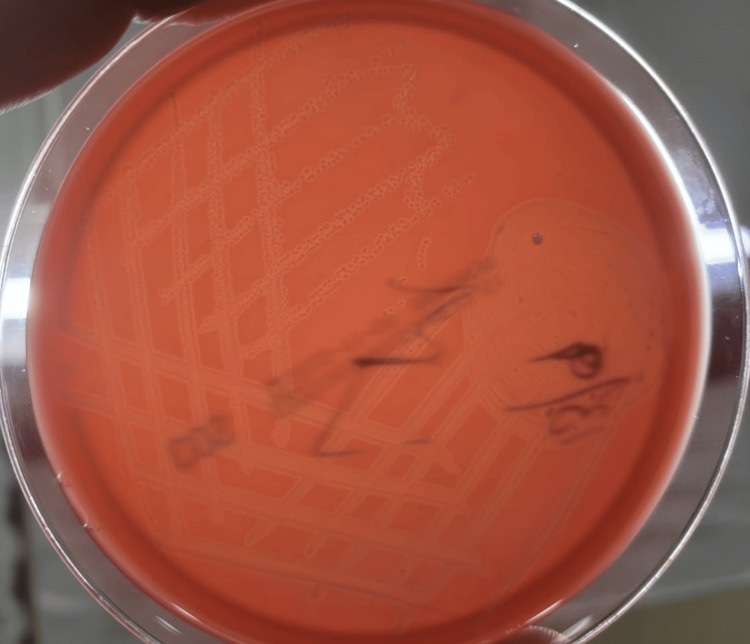
Listeria monocytogenes showing beta hemolysis on 5% sheep blood agar Blood agar showing the narrow zone of beta-hemolytic, 1−2 mm round, convex, smooth, translucent gray-white colonies

Gram smear of CSF revealed non-sporing gram-positive bacilli. The sample was inoculated on 5% sheep blood agar, chocolate agar, and MacConkey agar, and incubated at 37 °C in a candle jar overnight. The bacterial growth displayed 1−2 mm round, convex, smooth, and translucent gray-white colonies with a narrow zone of beta hemolysis on 5% sheep blood agar (Figure 1).

**Figure 2:**
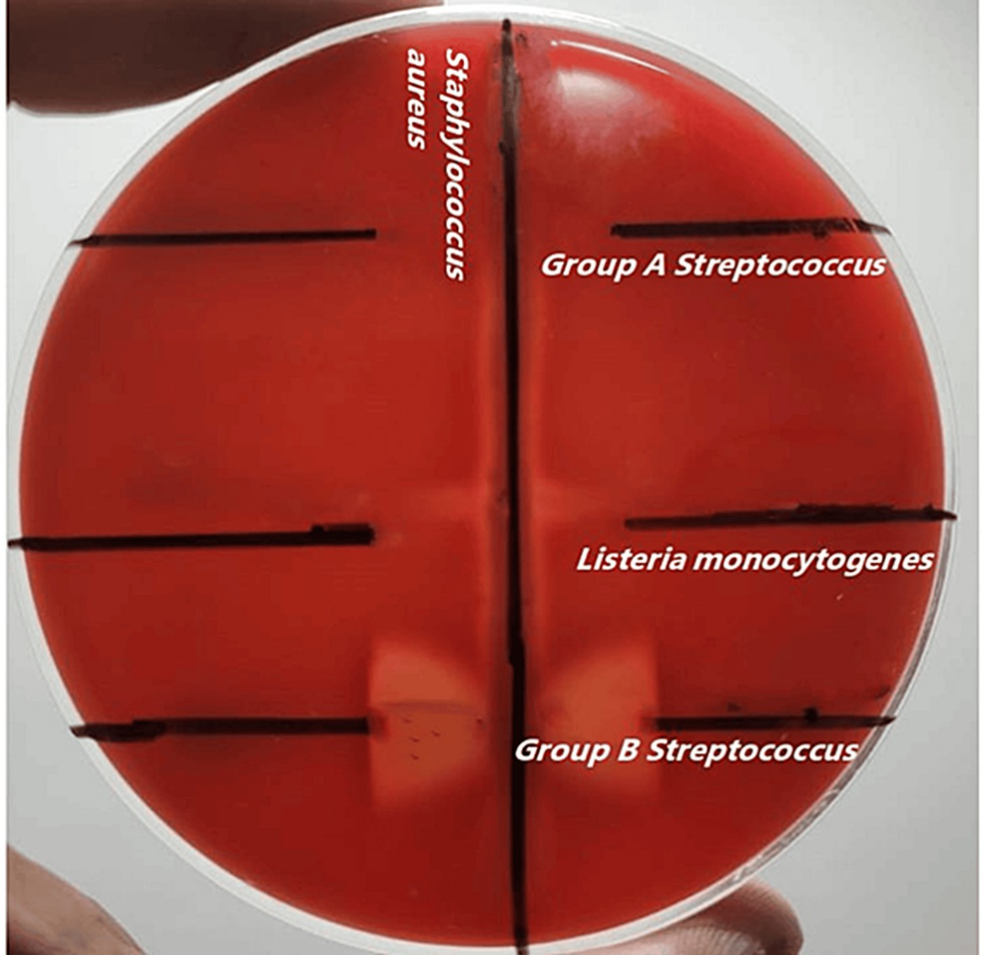
Christie, Atkins, Munch–Peterson-negative Listeria monocytogenes isolate The test isolate streaked in the center, not showing arrowhead hemolysis. *Group B Streptococcus* was used as the positive control and *Group A Streptococcus* was used as the negative control as shown in the figure.

Gram stain of the colonies from blood agar demonstrated Gram-positive bacilli with no spore or capsule. The catalase test performed from colonies was positive. Hanging drop from colonies on blood agar showed non-motile bacilli. Colonies were inoculated in peptone water for 4 hours at 25°C and 37 °C. Hanging drop from peptone water at 25°C showed tumbling motility while the one at 37 °C was non-motile. The isolate was negative for nitrate reductase and urease. It was oxidase negative. The isolate was the bile-esculin test positive. The organism showed fermentation of glucose and maltose, as well as L-rhamnose, but did not ferment D-xylose or mannitol. The Christie, Atkinson, Munch-Peterson (CAMP) test was negative for the isolate (Figure 2).

The isolate was confirmed to be *Listeria monocytogenes* by matrix-assisted laser desorption ionization-time of flight mass spectrometry (MALDI-TOF MS). Subsequently, the isolate was sequenced for the 16 S ribosomal gene and confirmed as *Listeria monocytogenes* and the sequence was submitted to GenBank and assigned accession no. OP600476.

**Figure 3:**
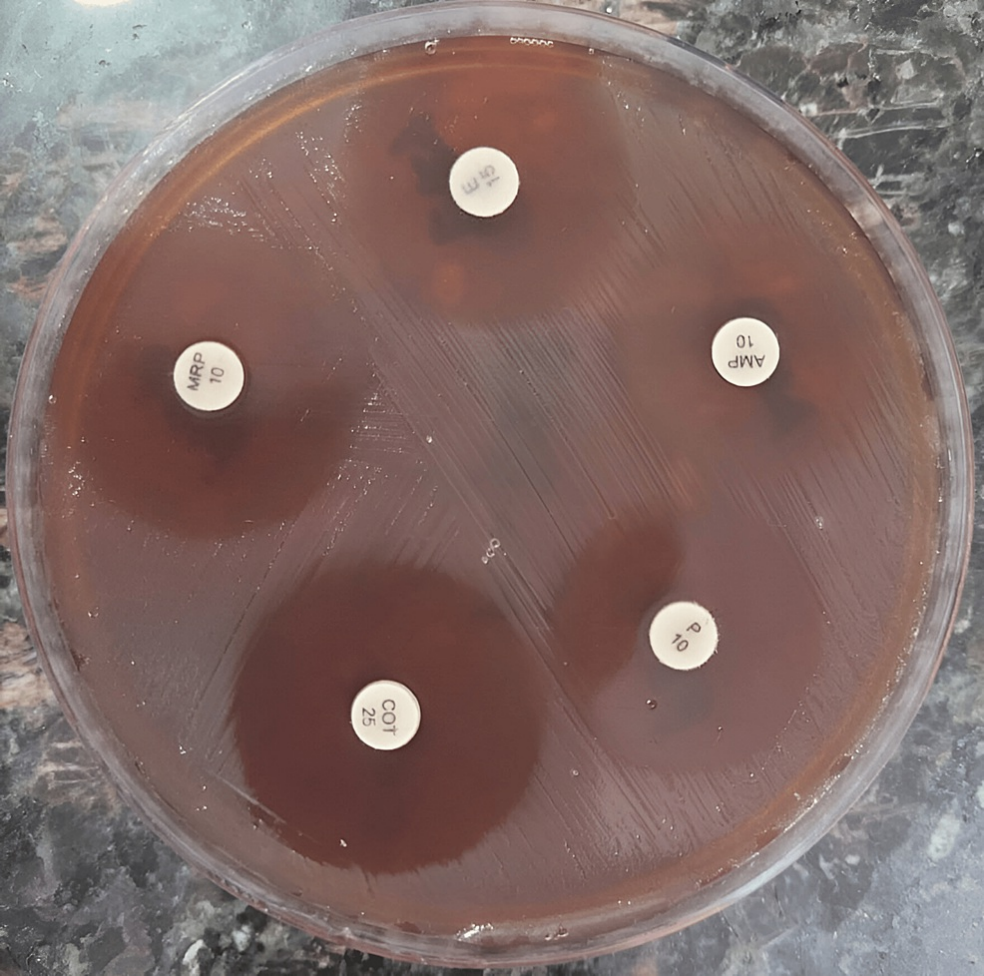
Kirby Bauer's disk-diffusion method of antimicrobial susceptibility testing of Listeria monocytogenes Isolate showing sensitive zones to ampicillin, penicillin, co-trimoxazole, erythromycin, and meropenem

Antimicrobial sensitivity was performed by Kirby Bauer's disk-diffusion method on blood agar. The organism was found to be sensitive to ampicillin, penicillin, co-trimoxazole, erythromycin, and meropenem by the European Committee on Antimicrobial Susceptibility Testing (EUCAST) guidelines (Figure 3) [7].

Blood culture showed no growth after 5 days of incubation at 37 °C.

On the ninth day of life, the infant was placed on bubble continuous positive airway pressure (CPAP) due to inadequate oxygen saturation while breathing room air and exhibiting reduced spontaneous response. In response to the declining saturation levels, the baby was intubated with an endotracheal tube (cuff size 3.5 mm) and initiated on mechanical ventilation.

Subsequently, the baby’s condition worsened, leading to the administration of IV meropenem (80 mg TDS) for treatment. The infant also experienced seizures, prompting the use of IV phenobarbitone (55 mg over 30 minutes). As ventilator support became necessary, the baby was transitioned to a bag and tube mechanical ventilator and then referred to another medical center due to ventilator unavailability.

At the new center, the baby was extubated and placed back on CPAP. After 24 hours, CPAP was discontinued, and the baby maintained adequate oxygen saturation on room air, eventually leading to discharge after complete recovery.

Case 2

An 11-day-old neonate, born full-term but with low birth weight and small for gestational age (SGA), weighing 2.2 kg, was admitted to the NICU at a tertiary care hospital. The baby presented with symptoms of loose stool lasting for two days, characterized by watery consistency and no associated blood. Additionally, the infant had a fever persisting for two days and was refusing breast milk for one day. The baby maintained a urinary output of three to four times a day.

**Figure 4:**
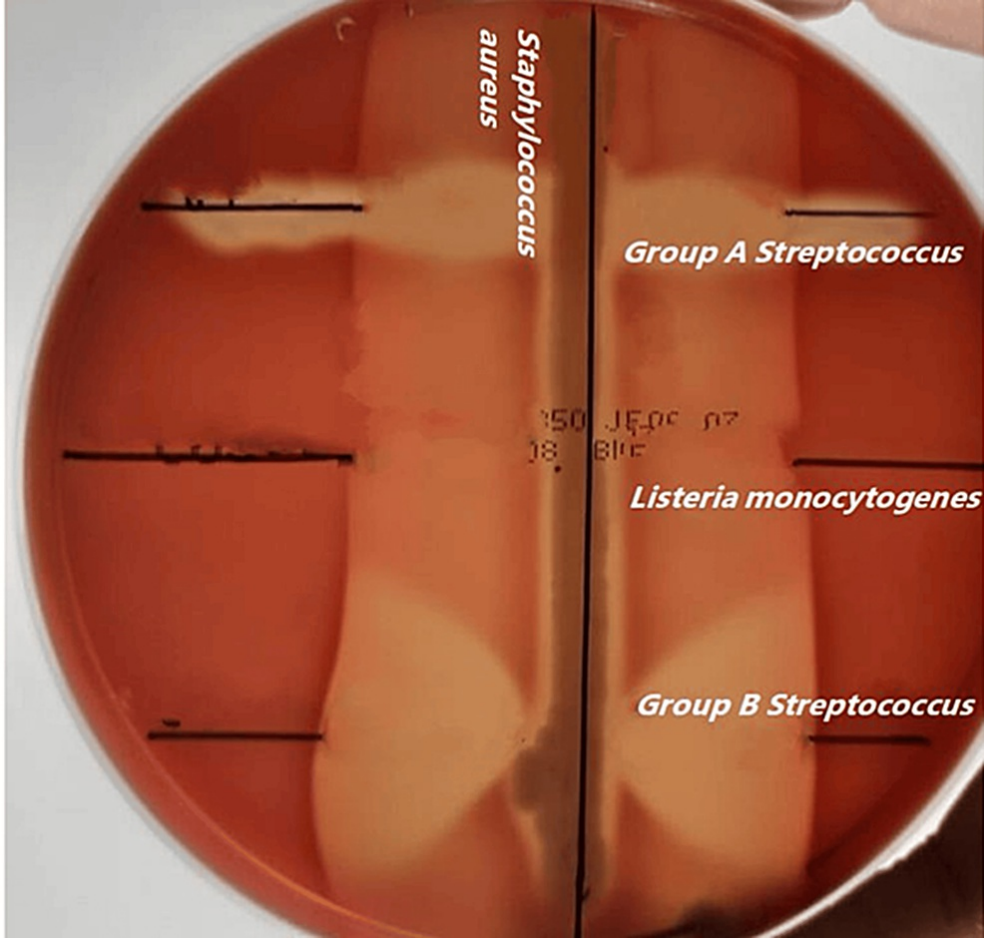
Christie, Atkins, Munch–Peterson-negative Listeria monocytogenes isolate The test isolate streaked in the center, not showing arrow head hemolysis. *Group B Streptococcus* was used as the positive control and *Group A Streptococcus* was used as the negative control in the test.

On examination, the heart rate, respiratory rate, and body temperature were 180/minute, 80/minute, and 103.8 °F, respectively. Peripheral pulse was feeble. Random blood sugar was 101 mg/dL. The baby was lethargic, dull, and not active. The baby was not icteric. In view of impending sepsis and meningitis, fluid boluses were given, and inotropic support was started. IV phenobarbitone 42 mg over 30 minutes was also given. Subsequently, there was a sudden episode of desaturation with poor respiratory effort. Bag and mask support was given but the child was unstable. Subsequently, the baby was intubated and put on mechanical ventilation. The child developed oral, nasal, and endotracheal bleeding. Packed RBC was transfused. The coagulation profile revealed coagulopathy. Fresh frozen plasma was transfused and IV vitamin K 1 mg OD was given. CSF and blood cultures were sent and the baby was empirically started on IV piperacillin-tazobactam 220 mg TDS and IV amikacin 33 mg OD. The CSF grew *Listeria monocytogenes*, which was identified biochemically and further confirmed by MALDI-TOF MS. The Christie Atkinson Munch-Peterson (CAMP) test was negative for the isolate (Figure 4).

The isolate was subsequently sequenced for the 16 S ribosomal gene and confirmed as *Listeria monocytogenes* and the sequence was submitted to GenBank and assigned accession no. OR272347. Blood cultures remained sterile after five days of incubation at 37 °C. Antimicrobial sensitivity was performed by Kirby Bauer's disk-diffusion method on blood agar. The organism was found to be sensitive to ampicillin, penicillin, co-trimoxazole, erythromycin, and meropenem by EUCAST guidelines [7]. The baby was started on IV meropenem (80 mg TDS) for treatment and the condition improved after continuous efforts and subsequently discharged from the NICU after three weeks.

## Discussion

The presence of Gram-positive bacilli in blood or CSF from neonates should not be dismissed as contaminants unless Listeria is definitively ruled out [8]. The organism may be mistaken for a contaminating non-pathogenic diphtheroid [9]. Reported analyses of a variety of foods for Listeria such as milk products, meat products, seafood, and vegetables have been shown to be contaminated with Listeria in India [10]. Listeriosis during pregnancy is a serious concern. It can lead to various complications, including miscarriage, stillbirth, premature delivery, and the birth of a low-birth-weight infant. The bacterium responsible for listeriosis, *Listeria monocytogenes*, has the ability to cross barriers such as the intestine, blood-brain, and placental barriers. Its virulence lies in its ability to thrive at low temperatures and even form biofilms [11].

In our cases, we could not assess the real cause of this neonatal listeriosis. In our cases, since the neonates were empirically started on broad-spectrum antibiotics, there was a significant improvement in their condition and they were saved. Both the cases discussed were CAMP-negative. This finding emphasizes that laboratories that depend only on conventional biochemical tests might miss these important pathogens. CAMP-negative *Listeria monocytogenes* is a rare phenomenon. This is the first case report of CAMP-negative Listeria from India. In a South African study conducted by Thomas TSM et al. in 2022, *Listeria monocytogenes* was isolated from food products, and 18% of the isolates tested negative in the CAMP test [12]. Another study by Fernandez-Garayzabal JF et al. in 1996 reported that 1.6% of *Listeria monocytogenes* isolates were also CAMP test negative [13].

## Conclusions

In our cases, we observed that *Listeria monocytogenes* may yield a negative CAMP test. Hence, relying solely on conventional biochemical tests may lead to a misdiagnosis of *Listeria monocytogenes*. To ensure accurate diagnosis, it is recommended to employ advanced bacterial identification techniques such as Vitek 2, MALDI-TOF MS, and 16S RNA sequencing. The Gram-positive bacilli isolated from CSF and blood are often dismissed as contaminants from the skin. However, an accurate diagnosis of listeriosis and the prompt initiation of treatment can be crucial for saving the lives of neonates.
